# Protocol: Adeno-Associated Virus-Mediated Gene Transfer in Ex Vivo Cultured Embryonic Mammary Gland

**DOI:** 10.1007/s10911-020-09461-4

**Published:** 2020-10-02

**Authors:** Qiang Lan, Marja L. Mikkola

**Affiliations:** Cell and tissue dynamics research program, Institute of Biotechnology, Helsinki Institute of Life Science (HiLIFE), University of Helsinki, Helsinki, Finland

**Keywords:** Organ culture, Ex vivo, Explant culture, Adeno-associated virus

## Abstract

Branching morphogenesis of the murine mammary gland starts during late embryogenesis. It is regulated by the signals emanating both from the epithelium and the mesenchyme, yet the molecular mechanisms regulating this process remain poorly understood. We have previously developed a unique whole organ culture technique for embryonic mammary glands, which provides a powerful tool to monitor and manipulate branching morphogenesis ex vivo. Nowadays, RNA sequencing and other transcriptional profiling techniques provide robust methods to identify components of gene regulatory networks driving branching morphogenesis. However, validation of the candidate genes still mainly depends on the use of the transgenic mouse models, especially in mammary gland studies. By comparing different serotypes of recombinant adeno-associated virus (rAAVs), we found out that rAAVs provide sufficient efficiency for gene transfer with different tissue preferences depending on the serotypes of the virus. AAV-2 and AAV-8 preferentially target epithelial and mesenchymal compartments, respectively, while AAV-9 infects both tissues. Here, we describe a protocol for AAV-mediated gene transfer in ex vivo cultured murine embryonic mammary gland facilitating gene function studies on mammary gland branching morphogenesis.

**Electronic supplementary material:**

The online version of this article (10.1007/s10911-020-09461-4) contains supplementary material, which is available to authorized users.

## Introduction

The embryonic development of the mammary gland in mouse starts at embryonic day (E) 10 with the formation of milk lines, which are two discontinued stripes of ventral lateral surface ectoderm between the fore and hind limb [1–3]. One day later, 5 pairs of symmetrically positioned mammary rudiments named placodes have formed, presumably by cell migration [4, 5]. By E13, placodes have extended into the underlying mesenchyme to form buds and are now surrounded by a condensed, mammary-specific mesenchyme [6, 7]. Starting from E15-E16, depending on the strain of mouse, the mammary bud starts sprouting and invades into the underlying fat pad, the precursor of adult mammary gland stroma. By E18.5 (just before birth), the mammary gland forms a small ductal tree with about 10–15 branches. After birth, mammary gland development is slow until the onset of the puberty. The interaction between mesenchymal and epithelial cells is essential for mammary gland morphogenesis at all developmental stages.

Due to the technical difficulties in studying the mammary branching morphogenesis and epithelial-mesenchymal interactions in vivo, several ex vivo culture methods have been developed. For example, epithelial organoids are cultured in various 3-dimensional (3D) matrices, but typically these cultures lack stromal cells, and hence the crucial epithelial-stromal crosstalk known to be essential for normal development [8, 9]. Organ culture is a powerful method widely used in developmental biology studies. We have previously established a whole organ culture technique as an alternative for organoid cultures, where embryonic mammary buds are microdissected with the surrounding stroma and cultured ex vivo [10, 11]. This method was initially developed in the 60’s [12], but it has not been used much by the scientific community ever since, likely due to technical challenges. We have developed this technique further, and now we routinely culture embryonic mammary glands from E12.5 to E13.5 for 3–7, even up to 9 days. During this time, the ductal system forms, it invades the stroma and branches essentially as in vivo. This system allows us to monitor and to manipulate (e.g. growth factors or drugs can be added and washed away) ductal invasion ex vivo.

Genetically modified mice remain one of the most widely used tools for analyzing gene function in mammalian development. However, the generation of genetically modified mice is costly and time-consuming, which severely limits their applications, especially in the post-genome era. Thus, gene modification in ex vivo cultured organs would be an ideal technique for gene function analysis before in vivo analysis of transgenic animals, especially when growth factors or drugs are not available. Although siRNA transfection has been successfully applied in ex vivo cultured embryonic kidneys [13] and salivary glands [14] already nearly two decades ago, their use has not been wide-spread. Furthermore, at least in our hands, epithelial cells of the embryonic mammary glands are refractory to transfection reagents such as Lipofectamine 3000 (Invitrogen), Lipofectamine RNAiMAX (Invitrogen) and HiPerFect (QIAGEN) in whole organ cultures (unpublished observations). Hsu et al. have compared different virus-mediated gene transfer methods in ex vivo cultured salivary glands and observed virus-type dependent selective targeting to epithelial or mesenchymal cells [15]. This pioneering study encouraged us to look for a feasible gene transfer method in the ex vivo cultured embryonic mammary gland. Here, we describe a protocol for recombinant adeno-associated virus (rAAVs)-mediated gene transfer method in ex vivo cultured murine embryonic mammary gland facilitating gene function studies during mammary gland branching morphogenesis.

## Reagents, Solutions and Materials

1. Sterile Dulbecco’s phosphate-buffered saline (DPBS, D5652, Sigma-Aldrich)
2. Sterile Phosphate-buffered saline (PBS; P4417, Sigma-Aldrich)
3. F-12/DMEM culture medium: Prepare the medium by mixing F-12-GlutaMAX (31765, Thermo Fisher) with DMEM-GlutaMAX (651965, ThermoFisher) at a 1:1 ratio and supplement with 10% (vol/vol) heat-inactivated fetal bovine serum (FBS; SV30160.03, HyClone/Thermo Fisher) and 20 U/ml penicillin-streptomycin (PS; 15140, Gibco/ Thermo Fisher)
4. Ascorbic acid (A4544, Sigma-Aldrich). 100 mg/ml stock solution is prepared by reconstitution in water and then filter-sterilized with a 0.22 μm filter (SCGPCARE, Millipore/Merck). The solution is divided into single-use aliquots and store at −20 °C and protected from light. Dilute 1:1000 in F12/DMEM culture medium right before use.
5. Dispase II (4942078001, Roche). Dissolve the powder to a final concentration of 1.25 U/ml in PBS and filter-sterilize with a 0.22 μm filter. Always use a freshly made working solution.
6. Thyrode’s solution (pH 7.4). Dissolve the following chemicals into 900 ml of MilliQ water:
  8.0 g NaCl (S5886, Sigma-Aldrich)
  0.2 g KCl (P5405, Sigma-Aldrich)
  0.05 g NaH_2_PO_4_ + H_2_O (S3522, Sigma-Aldrich)
  1.0 g D-(+)-Glucose (G7021, Sigma-Aldrich)
  1.0 g NaHCO_3_ (S5761, Sigma-Aldrich)

Adjust the pH to 7.4 and add MilliQ water to a final volume of 1000 ml. The solution is filter-sterilized with a 0.22 μm filter and stored at 4 °C for use.

**Fig. 1:**
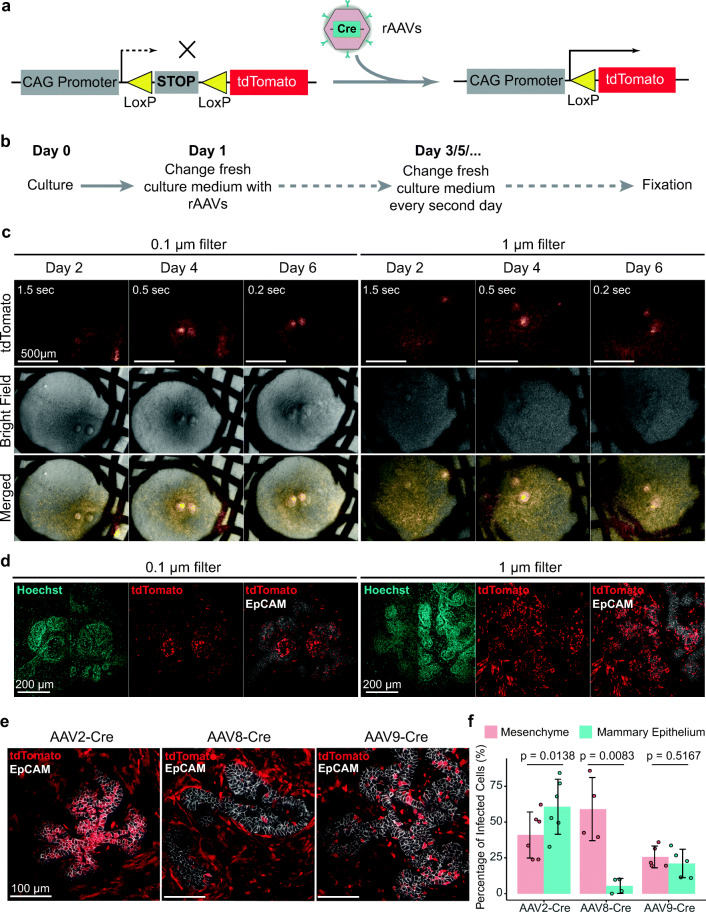
**Virus infection on the whole explant**. **a.** Schematic diagram illustrating the model used in this protocol. **b.** Scheme illustrating the schedule of the experiment. **c-d.** The filter with 1.0 μm pore size shows higher rAAVs infection efficiency compared with 0.1 μm. The explants were incubated in culture medium containing 1.4 × 10^9^ vg (virus genome)/ml AAV2-Cre (which harbors Cre recombinase) for 2 days. All the progenies of the infected cells will be labelled with tdTomato expression. The images were taken with stereomicroscope on day 2, 4 and 6 to monitor the growth of mammary buds and the expression of tdTomato reporter genes (**c**). The exposure time of tdTomato expression has been labelled in the image. The virus infection efficiency has been further confirmed by whole mount fluorescent immunostaining with anti-EpCAM antibody and Hoechst 33342 to visualize the epithelial cells and nuclei, respectively, and imaged with Zeiss LSM700 confocal microscope and the representative images of optical section were shown (d). **e-f**. Different serotype of rAAVs preferentially target different tissues. The explants were incubated in culture medium containing 8.6 × 10^9^ vg/ml AAV2-Cre, AAV8-Cre or AAV9-Cre, respectively, for 2 days. 7 days after culture, the virus infection efficiency was further confirmed by whole mount fluorescent immunostaining and confocal microscope and the representative images of optical section were shown (**e**). The infection efficiency in mammary epithelium and mesenchyme compartment from the same explant was determined separately by quantifying the progenies of infected and non-infected cells using Imaris 9.2 software (Bitplane). The figure was produced with R, a free software environment available at http://www.r-project.org/, and ggplot2 package [18] (**f**). The data are presented as mean ± SD. Statistical comparisons were performed by paired Student’s t test

7. 10x Pancreatin stock solution. Dissolve 0.85 g NaCl (S5886, Sigma-Aldrich) and 2.5 g Pancreatin (P3292, Sigma-Aldrich) into 100 ml of MilliQ water on a magnetic stirrer on ice for 3–4 h (or at 4 °C o/n). Remove the insoluble impurities by centrifugation at 5000 rpm for 10 min and filter-sterilize the supernatant with a 0.22 μm filter (usually at least two 0.22 μm filters will be needed to pass a volume of 100 ml due to the viscosity). Divide the solution into 1 ml aliquots (The stock solution can be used at least for 1 year when stored at −20 °C).
8. Pancreatin-trypsin working solution (pH 7.4). Dissolve 0.225 g of trypsin (T4799, Sigma-Aldrich/Merck) into 6-ml of Thyrode’s solution using a magnetic stirrer on ice. Add 1 ml of 10X pancreatin stock solution and 20 μl of PS (10,000 U/ml in stock) and adjust pH to 7.4 with NaOH. Adjust the volume to 10 ml with Thyrode’s solution and filter-sterilize with a 0.22 μm filter. Divide the solution into 1 ml aliquots and store at −20 °C. The working solution is stable for 2–3 weeks at −20 °C. Do not re-freeze the solution after thawing.
9. Nuclepore polycarbonate filters (pore size: 1 μm. 110610, Whatman). Sterilize and store the filters in 70% ethanol at room temperature. Wash the filter with sterilized PBS inside laminar hood three times and cut the filter to approximately 5 mm squares in PBS in advance. The filter squares can be stored in PBS at 4 °C for 1–2 weeks.
10. 35 mm plastic Petri dishes (121V, Sterilin/Thermo Scientific)
11. Glass Pasteur pipettes
12. Horizontal laminar flow hood
13. Stereo microscope with camera
14. Metal grids: Corrosion-resistant stainless-steel mesh (0.7 mm mesh size) is cut into approximately 30 mm diameter disks and the edges are bent to reach about 3 mm height. Make small holes (e.g. 2 rows of 3) into the grid to facilitate the observation of the explants. Grids can be washed and autoclaved multiple times. Alternatively, commercial cell culture inserts could be used to replace the grid and Nuclepore filters.
15. Two disposable 26-gauge (303800, BD Microlance) or 25-gauge (300600, BD Microlance) needles attached to 1 ml plunger-removed disposable plastic syringes (303172, BD Plastipak).
16. Recombinant adeno-associated virus (rAAVs). Different serotypes of rAAVs (AAV-2, AAV-8 and AAV-9) were purchased from AAV Gene Transfer and Cell Therapy Core Facility, Faculty of Medicine, University of Helsinki. The virus particles were produced with viral vector pSub-CAG-WPRE-Cre and corresponding packaging vectors.
17. Virkon (LANXESS). Dissolve the powder in water. 1% (m/v) for surface disinfection. In order to disinfect the liquid waste, mix with 5% (m/v) Virkon with 1:1 ratio in a sealed container and incubate in room temperature for 15 min.
18. (Optional) tdTomato reporter mouse (007914, The Jackson Laboratory). In order to better illustrate the virus infection efficiency, we used tdTomato Cre reporter mouse [16] in this protocol. When crossed with a wildtype mouse, all the offspring carry one allele of tdTomato reporter gene, which contains a floxed stop cassette before the coding region and prevents the expression of the gene. After rAAV-Cre infection, the Cre recombinase translated from the viral genome will induce DNA recombination to remove the stop cassette in the virus-infected cells and initiate the expression of tdTomato (Fig. 1a).
19. (Optional) 4% PFA (Parafomaldehyde) in PBS (multiple suppliers).
20. (Optional) 0.3% PBST. 0.3% (vol/vol) Triton X-100 (04807423, MP Biomedicals) in PBS.
21. (Optional) Blocking solution. 5% (vol/vol) goat serum (16210064, Gibco/Thermo Fisher), 0.5% (mass/vol) BSA (A2153, Sigma-Aldrich) in 0.3% PBST.
22. (Optional) Confocal microscope. Zeiss LSM700 or Leica SP8 with suitable laser source.
23. (Optional) Rat Anti-Mouse CD326 (EpCAM) antibody (552370, BD Pharmingen, 1:2000).
24. (Optional) Goat anti-Rat IgG, Alexa Fluor 647 (A-21247, Invitrogen/ThermoFisher Scientific, 1:1000).
25. (Optional) Hoechst 33342 (H3570, Invitrogen/ThermoFisher Scientific, 1:1000)
26. (Optional) Mounting Medium (H-1000, Vector Laboratories).

## Method

### Mammary Tissue Dissection

1. **(Day 0)** The mammary tissue is dissected as previously described [11]. Briefly, E13-E13.5 mouse embryos are removed from the uterus and dissected in DPBS. The flank skin of the mammary region is carefully dissected out with small scissors. All the explants are transferred to a 35 mm plastic Petri dish using a Pasteur pipette and proceed to step 2.

### Separation of the Skin Epithelium from the Mesenchyme

The separation procedure is modified from the protocol previously described [11] to facilitate the process.

2. Replace DPBS with 2 ml freshly prepared 1.25 U/ml Dispase II solution in a 35 mm plastic Petri dish.
3. Keep the Petri dish on a linear shaker mixer with speed around 40 rpm in 4 °C for 15–25 min, depending on the age of the embryos (longer treatment time for older embryos) and the activity of the enzymes. Check the explants under the stereomicroscope and proceed to step 4 when epithelium and mesenchyme appear separated at the edge of the explants (Note 1).
4. Replace the Dispase II solution with pancreatin-trypsin working solution and incubate for 4–5 min at room temperature. To aid the process, gently manually rotate the Petri dish horizontally. Monitor the tissues under the stereomicroscope to determine the optimal incubation time. Proceed to step 5 when a big piece of epithelium starts to release from the explants.
5. Replace the pancreatin-trypsin working solution with F-12/DMEM culture medium to inactivate the enzyme activity. Recover the tissue on ice for 30–45 min and protect from light if necessary.
6. After incubation, transfer the explants to a small glass Petri dish using a glass Pasteur pipette. Add enough fresh F-12/DMEM culture medium to cover all the explants and remove the skin epithelium by using needles with 1 ml plunge-removed syringes under the stereomicroscope.
7. Proceed to step 8a for virus infection of whole explants (option 1) or step 8b for virus infection of mammary buds in a hanging drop (option 2).

### Virus Infection of the Whole Explant (Option 1)

Virus infection can be performed directly on the whole cultured explants. The tissue culture is performed as previously described [11] with minor modifications.

- Place a sterilized metal grid on a 35 mm plastic Petri dish.
- Prepare the culture medium by adding ascorbic acid stock solution into F-12/DMEM culture medium to a final concentration of 100 μg/ml. Only use culture medium with a fresh supplement of ascorbic acid.
- Add the culture medium on top of the metal grid and avoid trapping any air bubbles underneath the grid.
- Transfer the explants with filter squares to the top of the metal grid. To get optimal rAAVs infection efficiency, use filters with pore size of 1.0 μm instead of 0.1 μm in the original protocol [11] (Fig. 1c-d) (Note 2).
- Culture tissues in a humidified incubator at 37 °C with an atmosphere of 5% CO_2_ and perform rAAVs infection the next day.
- **(Day 1)** Change 2 ml of F-12/DMEM culture medium freshly supplemented with ascorbic acid.
- Add rAAVs into the medium to optimal virus concentration (Note 3 and 8). From this step, all the disposables and waste solutions should be collected cautiously (Note 4). Depending on the purpose of the experimental design, different serotypes of rAAVs can be used. Although there are no exclusive differences, we found that AAV-2 and AAV-8 preferentially target epithelial and mesenchymal compartments, respectively, while AAV-9 infects both tissues (Fig. 1e-f) (Note 6).
- **(Day 3)** Two days later, change 2 ml/dish of fresh virus free F-12/DMEM culture medium freshly supplemented with ascorbic acid. Continue changing the medium every second day until sufficient branching events are observed.
- Proceed to step 20 for fixation and visualization.

### Virus Infection of Mammary Buds in a Hanging Drop (Option 2)

**Fig. 2:**
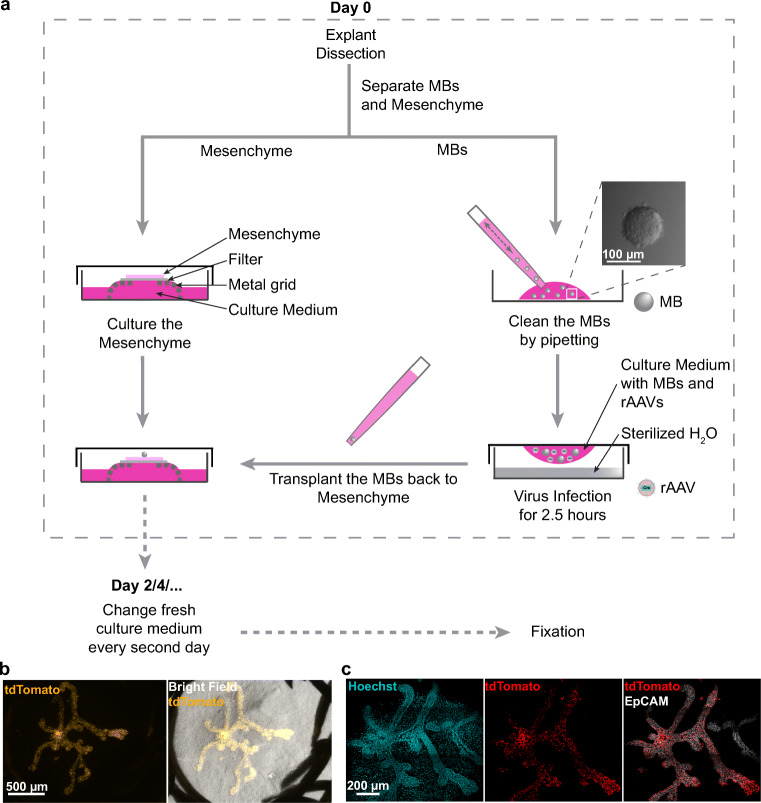
**Virus infection of mammary buds in a hanging drop**. **a.** Scheme illustrating the procedure and schedule of the experiment. **b-c.** Example of growing mammary buds (MBs) infected within 15 μl hanging drop containing 1.45 × 10^12^ vg/ml AAV8-Cre (stock in PBS). Images were taken 7 days after culture with stereomicroscope before fixation (b) or Leica SP8 confocal microscope after whole mount fluorescent immunostaining with EpCAM antibody and Hoechst 33342 and the representative images of optical section were shown (**c**)

In order to further increase the specificity of the infection in epithelial cells, an alternative method of virus infection is performed in isolated mammary buds (MBs) in a hanging drop (Fig. 2).

8b. **(Day 0)** Further dissect the MBs out from the mesenchyme with needles. Try to keep the mesenchyme as intact as possible (Online Resource 1: Fig. S1a-b).
9b. Collect the mesenchyme and perform normal culture as described in step 8a-12a.
10b. Remove the surrounding mesenchyme from the MBs as much as possible. Be cautious not to punch the MBs with the needle and discard the damaged MBs, as the MBs are very sensitive and will not grow if damaged.
11b. Collect the MBs to a new 35 mm plastic Petri dish using a small pipette with a 10 μl plastic pipette tip. Pre-coat the pipette tip with culture medium before use to avoid the MBs adhering to the surface of pipette tip.
12b. Gently pipette the MBs with fresh culture medium several times to further dissociate the MBs from the surrounding mesenchyme (Fig. 2a).
13b. Dilute the rAAVs to an optimal concentration with culture medium (Note 3 and 8). Starting from this step, all the disposables and waste solutions should be collected cautiously (Note 4).
14b. Collect the MBs to the cap of a new 35 mm plastic Petri dish in a drop (Fig. 2a). Replace the medium with 10–20 μl virus-containing medium and flip the cap to cover the Petri dish containing sterilized H_2_O to avoid drying of the drop. Incubate the Petri dish in the humidified incubator at 37 °C with an atmosphere of 5% CO_2_ for 2.5 h for infection (Note 5).
15b. Wash the MBs gently with fresh culture medium for 2–3 times by replacing the liquid.
16b. Use a small pipette with pre-coated 10 μl plastic pipette tip to transplant the MBs one by one to the top of the cultured mesenchyme from step 9b under the stereomicroscope. (Note 7, Online Resource 1: Fig. S1c-i)
  i. Adjust the volume setting for the pipette to 10 μl and pre-coat the plastic tip by aspirating 2–3 times F-12/DMEM culture medium.
  ii. Push the plunger in the air at the stop and place the tip into the fresh F-12/DMEM culture medium.
  iii. Release the plunger halfway to aspirate approximately 4–5 μl medium and then place the tip to the medium with virus infected MBs (Online Resource 1: Fig. S1c).
  iv. Release the plunger gently to aspirate one MB and pause immediately when the MB entered the tip (Online Resource 1: Fig. S1d-e).
  v. Hold the position of the plunger. If the pre-coating works well, the MB should not stick to the inner surface of the tip and you can see the MB entering the tip under the stereomicroscope. Move the tip out of the medium before the MB drops out from the tip and try to keep the MB at the opening of the tip.
  vi. Push the plunger a bit in the air to force the liquid surface out of the plastic tip without forming any drop. In this case, the MB will move closer to the opening of the tip.
  vii. Touch the mesenchyme with the tip gently without pushing the plunger and the MB will be stuck with the mesenchyme (Online Resource 1: Fig. S1f-i).
  viii. Repeat the above steps to transplant the MB one by one.
17b. Incubate the MBs with mesenchyme in the humidified incubator at 37 °C with an atmosphere of 5% CO_2_.
18b. **(Day 2)** Change 2 ml/dish of fresh virus-free F-12/DMEM culture medium freshly supplemented with ascorbic acid. Continue changing the medium every second day until sufficient branching events are observed (Fig. 2b).
19b. Proceed to step 20 for fixation and visualization.

### Fixation and Visualization

The explants can be fixed and processed for carmine alum staining, conventional histology or whole-mount immunofluorescence staining for confocal microscopy [11, 16, 17].

20. (Optional) Whole-mount immunofluorescence staining.
  i. Fix the samples in 4% PFA at 4 °C overnight with gentle shaking.
  ii. Wash the samples three times 20–30 min with PBS at room temperature (RT) on a shaker.
  iii. Permeabilize the samples with 0.3% PBST for two hours at RT.
  iv. Incubate the samples in blocking solution at RT for an hour.
  v. Remove the blocking solution and incubate the samples with antibody diluent consisting of EpCAM antibody (1:1000) and Hoechst 33342 (1:1000) in blocking solution at 4 °C with gentle shaking for 1–2 days.
  vi. Remove the antibody diluent and wash the sample three times 20–30 min with 0.3% PBST at RT.
  vii. Add secondary antibody dissolved in 0.3% PBST solution containing 1% BSA (mass/vol) and incubate at 4 °C overnight.
  viii. Remove the antibody diluent and wash the sample three times 20–30 min with 0.3% PBST at RT.
  ix. Wash the samples three times 20–30 min with PBS at RT.
  x. Incubate the samples with 4% PFA for 10 min at RT.
  xi. Remove the PFA and wash the sample three times 20–30 min with PBS at RT.
  xii. Store the samples in PBS at at 4 °C or mount slides with Vectashield Mounting Medium and a glass coverslip for imaging with confocal microscope.

## Notes

1. The enzymatic activity varies, and the duration of the treatment may be different depending on the batches, especially for pancreatic-trypsin treatment. Some of the mesenchyme may be lost during the treatment, therefore monitor the pancreatic-trypsin treatment frequently and inactivate the enzyme if large pieces of mesenchyme start detaching from the rest of the explant or the solution becomes very viscous. The separation will be more obvious during the recovery time in step 5.
2. The transparency of the filter paper with the pore size of 1.0 μm is lower than that of the 0.1 μm pore sized filter. Although the mammary gland is still visible, it is more difficult to monitor branching morphogenesis using transmitted light when using the 1.0 μm filter paper.
3. Depending on the supplier and serotype, the rAAVs can be delivered within different solutions, typically in PBS or iodixanol solution. In our test, high concentration of iodixanol solution inhibits the outgrowth of the cultured mammary gland, and a minimal 1:20 dilution of virus stock is needed to minimize the toxicity to an acceptable level.
4. Be cautious about the biosafety containment rules. Usually, the production of rAAVs must be handled at Biosafety Level 2 (BSL-2), but infection of rAAVs could be handled at BSL-1. However, if any hazardous transgene is included, BSL-2 containment rules apply. For disinfection, we use 1% Virkon solution for the surface and 1:1 mixture with 5% Virkon solution for waste liquid with 15–30 min incubation at room temperature.
5. Mesenchyme is crucial for keeping the identity of the mammary epithelium. In our test, mammary buds do not grow in mammary mesenchyme if they are incubated first in a hanging drop for a prolonged period of 3–4 h. Iodixanol solution coming with rAAVs stock decreases the time window further. To avoid this, try to use rAAVs with a high titer or delivered in PBS.
6. Several factors influence the expression level of a transgene carried by the virus genome, including the virus infection efficiency (which also influences the copy number of the virus genome in the cell) and the promoter used to drive transgene expression. Depending on the purpose of the experiment, selection of the right serotype of the rAAVs will significantly increase infection efficiency. However, for an optimal expression of a transgene in a specific tissue, the efficiency of different promoters should be further evaluated. For example, a transgene driven by keratinocyte-specific promoter, delivered in AAV2, is likely to specifically and efficiently express in mammary epithelial cells even using the whole explant infection method (option 1).
7. In order to avoid interaction of MBs close to each other, we normally transplant only one or two MBs to each cultured mesenchyme. If there are two, try to keep them far away from each other.
8. According to our experience, by using the whole explant infection method (option 1), 3.2 × 10^9^ to 8.6 × 10^9^ vg/ml AAV2-Cre or 8.6 × 10^9^ to 9.7 × 10^10^ vg/ml AAV9-Cre are able to infect around 20–50% epithelial cells. Meanwhile, 8.6 × 10^9^ vg/ml AAV8-Cre is able to infect around 50–60% mesenchymal cells. On the other hand, by using the hanging drop infection method (option 2), 1.45 × 10^12^ vg/ml AAV8-Cre or 6.77 × 10^11^ vg/ml AAV9-Cre are able to infect around 50–60% epithelial cells, which is much higher in concentration but less in total amount of virus that has been used in option 1. Due to the low titer of the AAV2-Cre virus available to us and toxicity of iodixanol solution, we were unable to apply AAV2-Cre to MBs (option 2) with sufficient efficiency without significantly compromising the outgrowth of the mammary gland. Based on our experience, we would suggest 5 × 10^10^ vg/ml AAV2-Cre as a starting point for the trial to target around 50% epithelial cells.

## Electronic supplementary material

Online Resource 1 **Fig. S1 Mammary bud dissection and transplantation. a-b.** Example images of an explant without the skin epidermis before (a) and after (b) removing the MBs. **c-e.** Image series demonstrating how to pick up a single MB for transplantation. A small pipette is used to gently place a culture medium pre-coated 10 μl plastic tip containing 4–5 μl medium into the medium with MBs (c). The plunger of the pipette is gently released to aspirate one MB and paused immediately after the MB has entered the tip (d). The plunger is carefully adjusted to keep the MB close to the opening of the tip (e). The tip is brought to the mesenchyme and the MB is released when gently touching the mesenchyme with the tip. **f-i.** Fluorescent (h and i) and bright field (f and g) images of the mammary mesenchyme before (f and h) and after (g and i) receiving the transplanted MB (green in i). Scale bar: 500 μm. (PDF 7307 kb)
