# Supplementary figures and images for: Protocol: Adeno-Associated Virus-Mediated Gene Transfer in Ex Vivo Cultured Embryonic Mammary Gland
Source: J Mammary Gland Biol Neoplasia. 2020 Oct 2;25(4):409–16. doi: 10.1007/s10911-020-09461-4 (PMC7960627; doi:10.1007/s10911-020-09461-4)

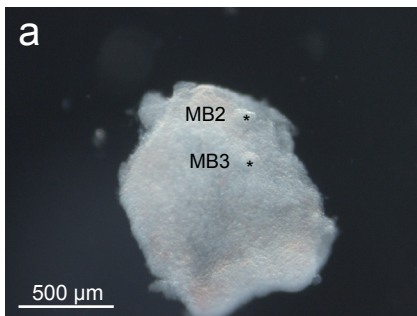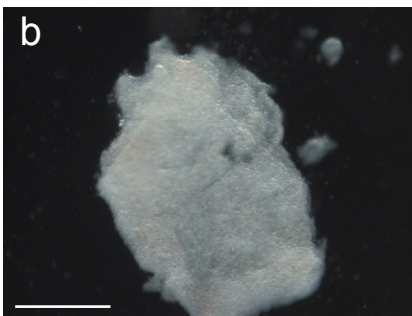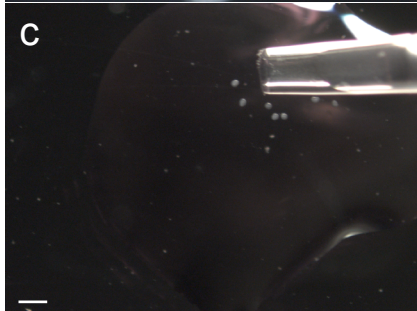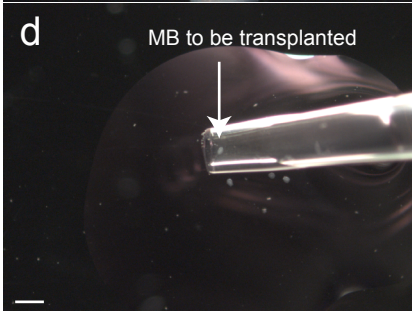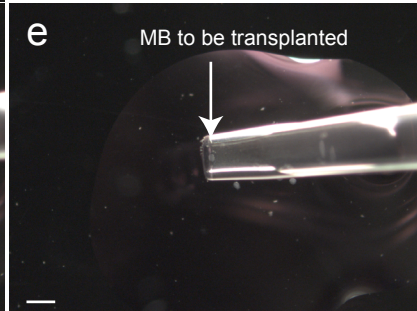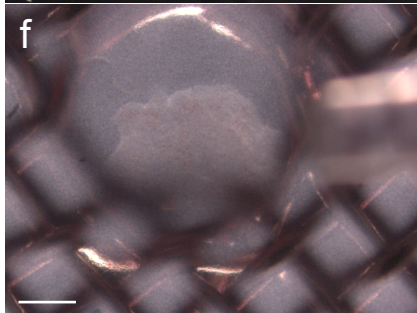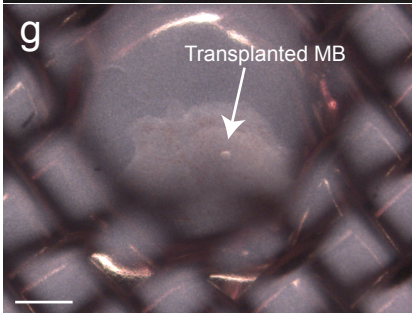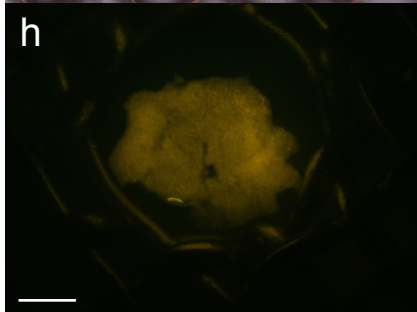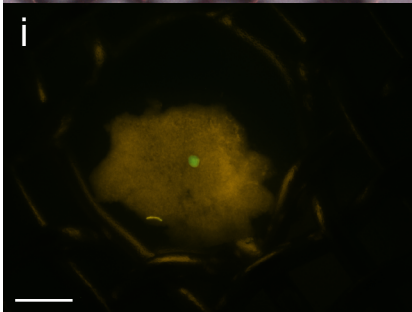

Supplement: Supplementary file 1 — Fig. S1 Mammary bud dissection and transplantation. a-b. Example images of an explant without the skin epidermis before (a) and after (b) removing the MBs. c-e. Image series demonstrating how to pick up a single MB for transplantation. A small pipette is used to gently place a culture medium pre-coated 10 μl plastic tip containing 4–5 μl medium into the medium with MBs (c). The plunger of the pipette is gently released to aspirate one MB and paused immediately after the MB has entered the tip (d). The plunger is carefully adjusted to keep the MB close to the opening of the tip (e). The tip is brought to the mesenchyme and the MB is released when gently touching the mesenchyme with the tip. f-i. Fluorescent (h and i) and bright field (f and g) images of the mammary mesenchyme before (f and h) and after (g and i) receiving the transplanted MB (green in i). Scale bar: 500 μm. (PDF 7307 kb) [file 10911_2020_9461_MOESM1_ESM.pdf]
